# Supplementary figures and images for: Influenza A virus NS1 protein hijacks YAP/TAZ to suppress TLR3-mediated innate immune response
Source: PLoS Pathog. 2022 May 3;18(5):e1010505. doi: 10.1371/journal.ppat.1010505 (PMC9122210; doi:10.1371/journal.ppat.1010505)

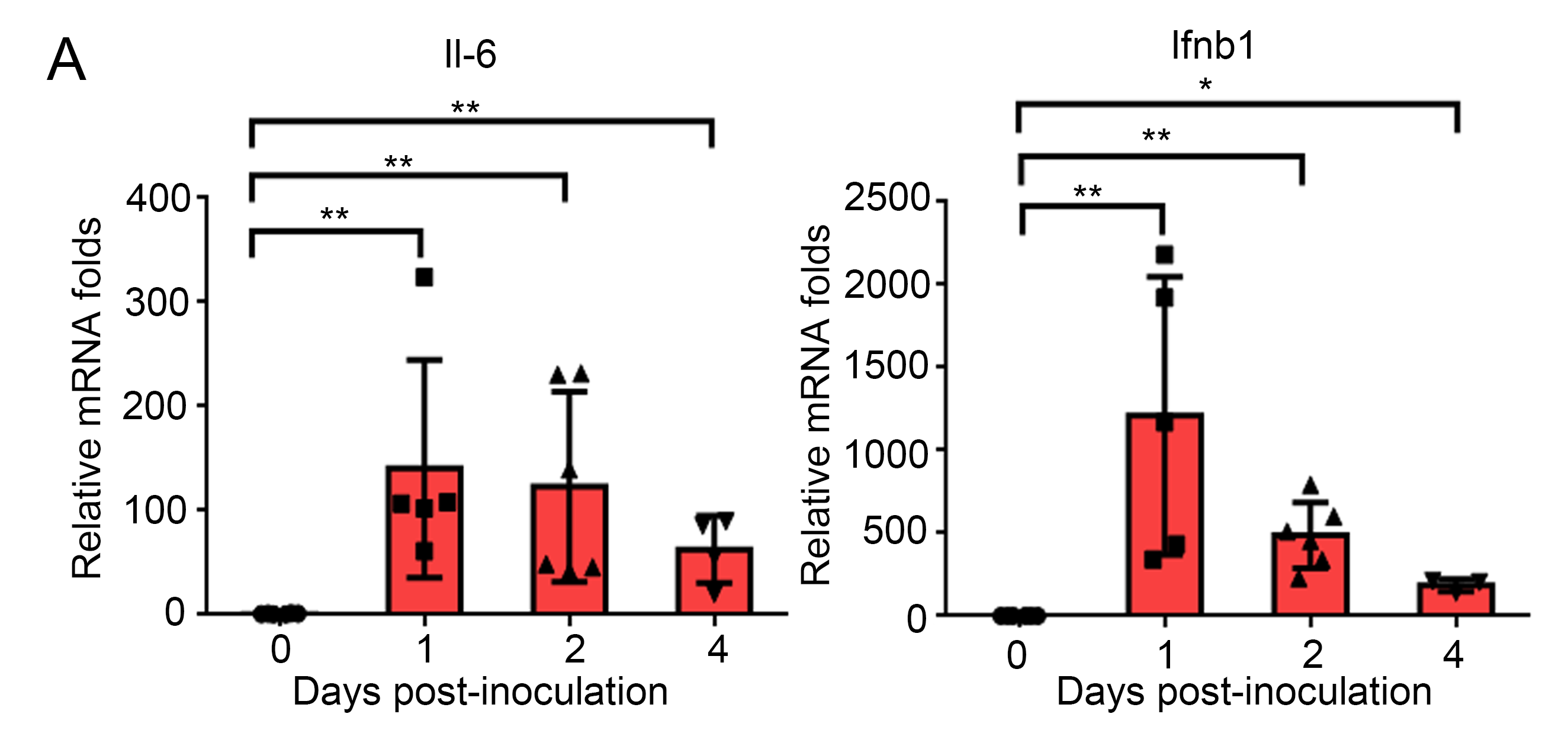

Supplement: S1 Fig — (A) Female C57BL/6J mice (n = 6 per group) were infected intranasally with 103 PFU of PR8. Lungs were harvested at different days post inoculation for analyzing changes in mRNA expression of Il-6 and Ifnb1. Data are presented as means ± SD. *p < 0.05, **p < 0.01, ***p < 0.001. (TIF) [file ppat.1010505.s001.tif]

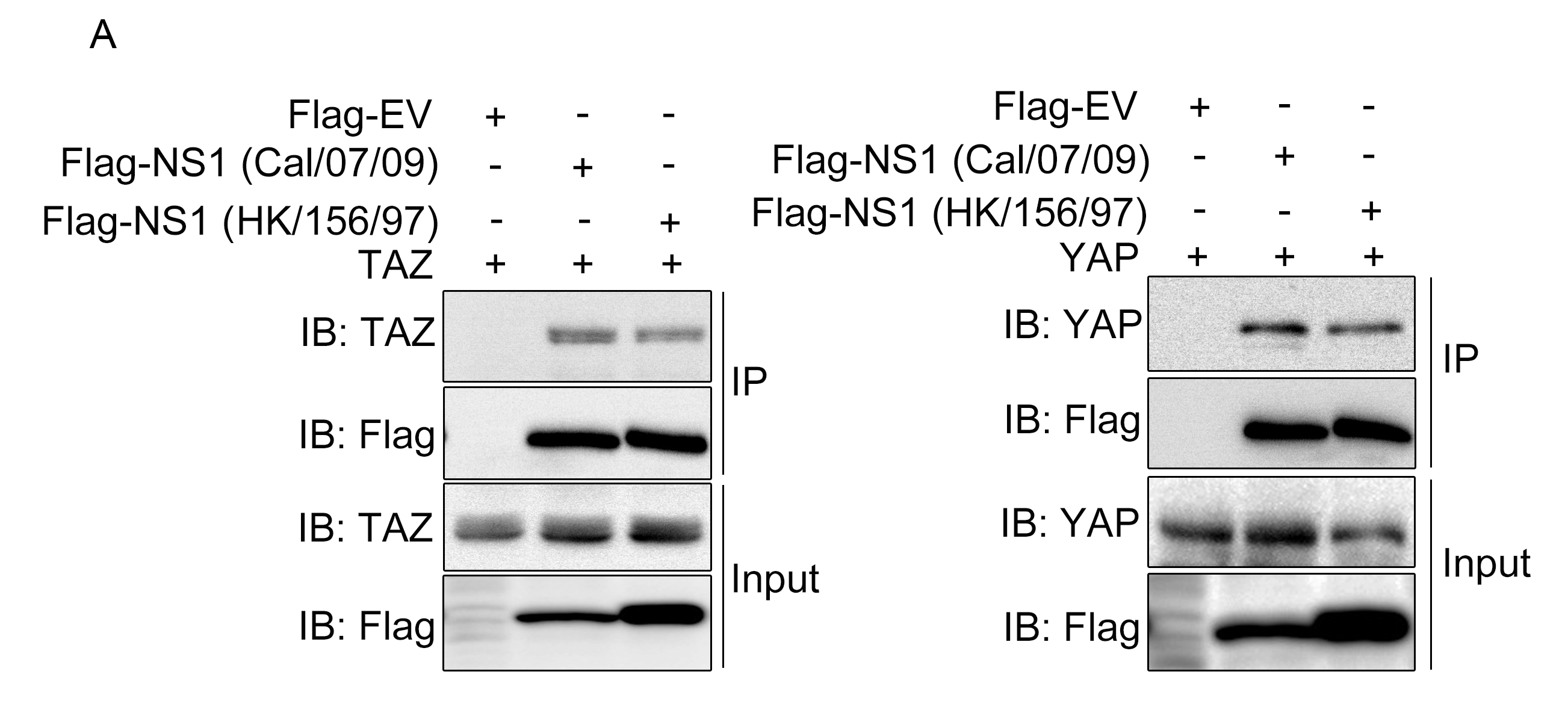

Supplement: S2 Fig — (A) 293T cells were co-transfected with TAZ or YAP plasmids, with Flag-EV and Flag-NS1 of the indicated IAV strains. WCL was subjected to immunoprecipitation with M2 beads. Bound proteins and input lysis were analyzed by immunoblotting with indicated antibodies. All of the experiments were repeated at least three times. (TIF) [file ppat.1010505.s002.tif]

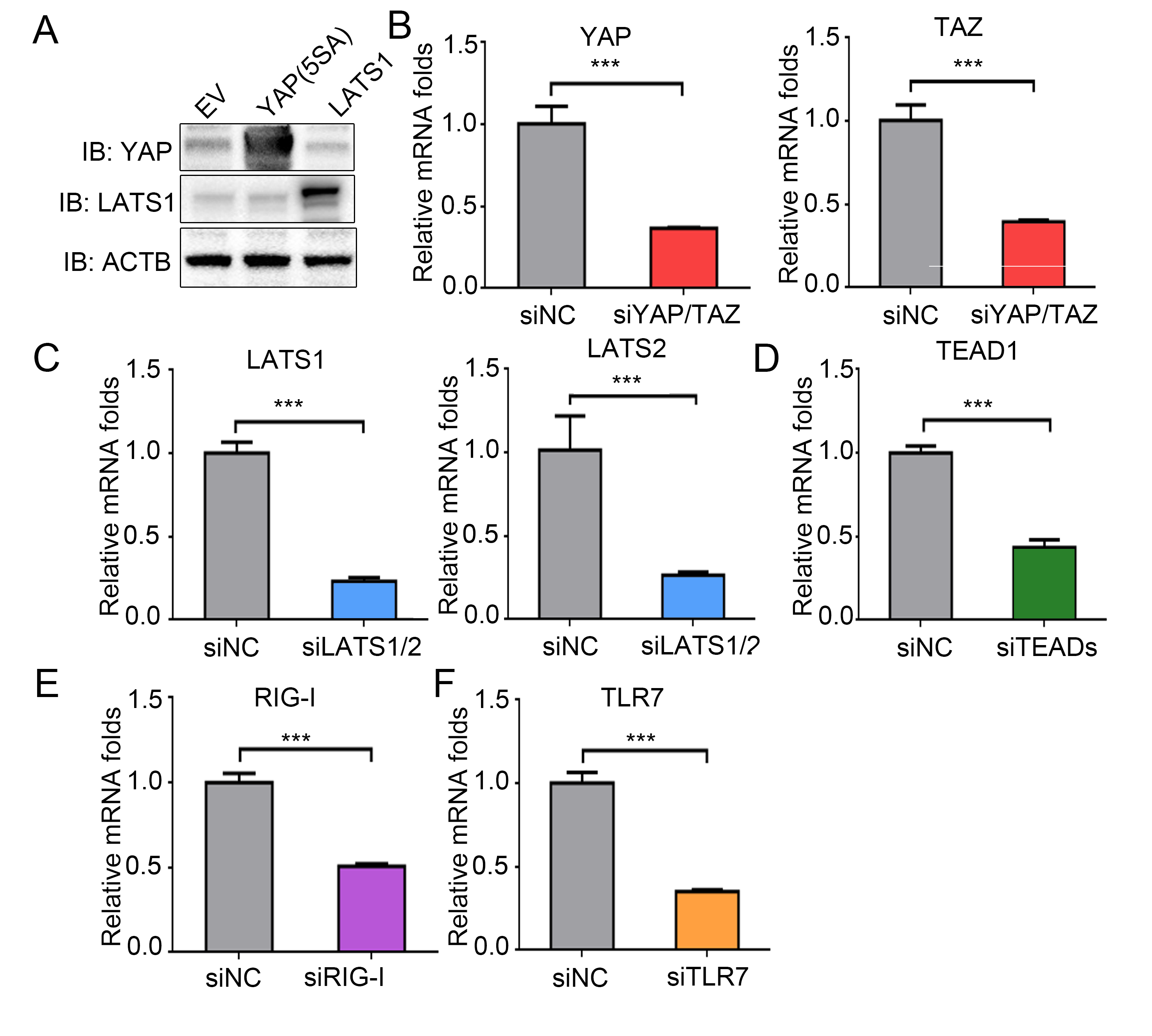

Supplement: S3 Fig — (A) The expression levels of stably expressed exogenous YAP(5SA) and LATS1 were determined by immunoblotting. (B-F) The changes in mRNA expressions of YAP/TAZ (B), LATS1/2 (C), TEAD1 (D), RIG-I (E) and TLR7 (F) in A549 cells transfected with corresponding siRNAs were analyzed by RT-qPCR. All of the experiments were repeated at least three times. Data in (B-F) are presented as means ± SD. *p < 0.05, **p < 0.01, ***p < 0.001. (TIF) [file ppat.1010505.s003.tif]

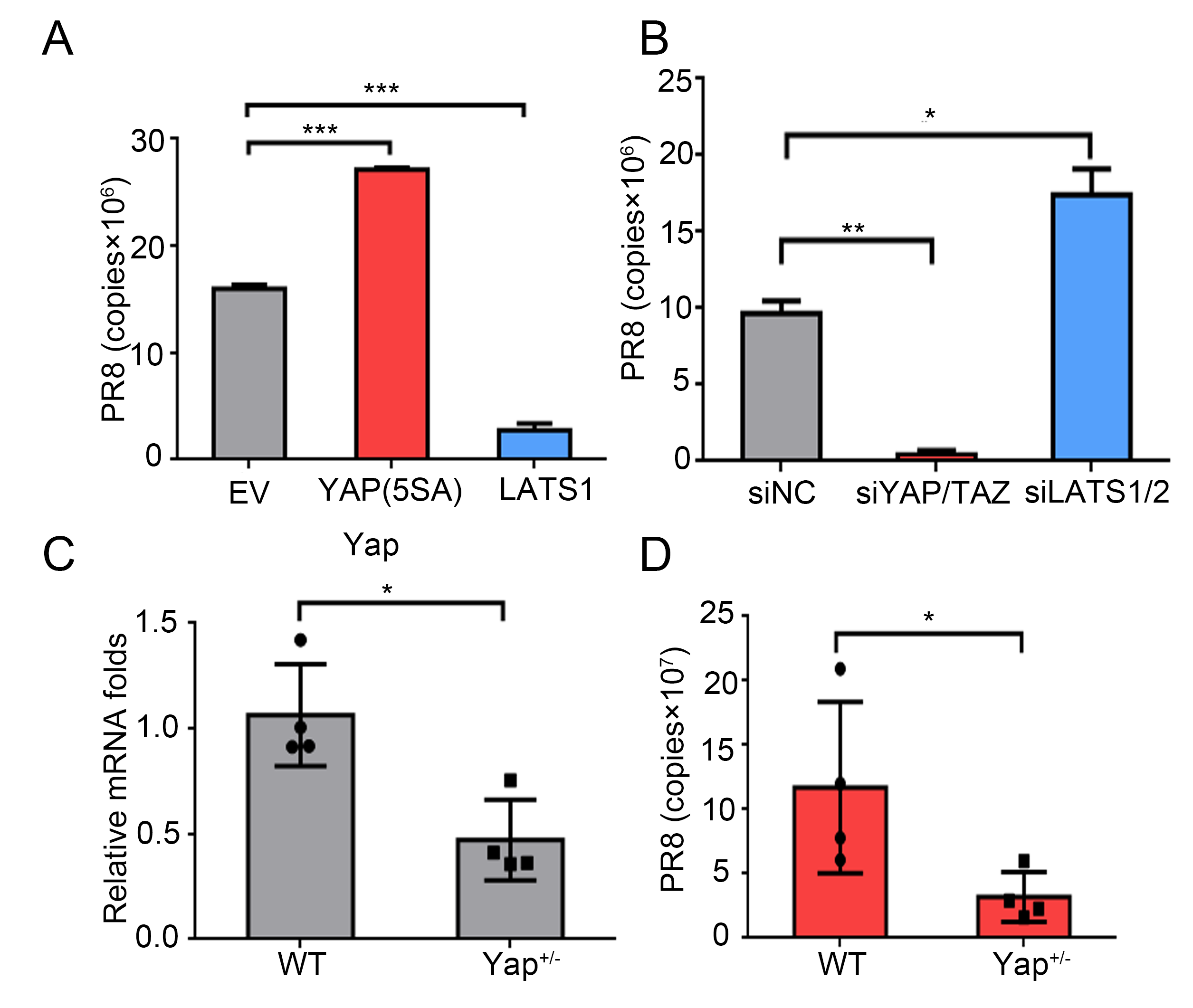

Supplement: S4 Fig — (A, B) Virus copies from cells transfected with indicated plasmids (A) or siRNAs (B) were determined by RT-qPCR. (C, D) The expression of Yap (C) and virus copies (D) in PR8-infected WT and Yap+/- mice (n = 4 per group) were assessed by RT-qPCR. Data in (A, B) were repeated at least three times. Data are presented as means ± SD. *p < 0.05, **p < 0.01, ***p < 0.001. (TIF) [file ppat.1010505.s004.tif]

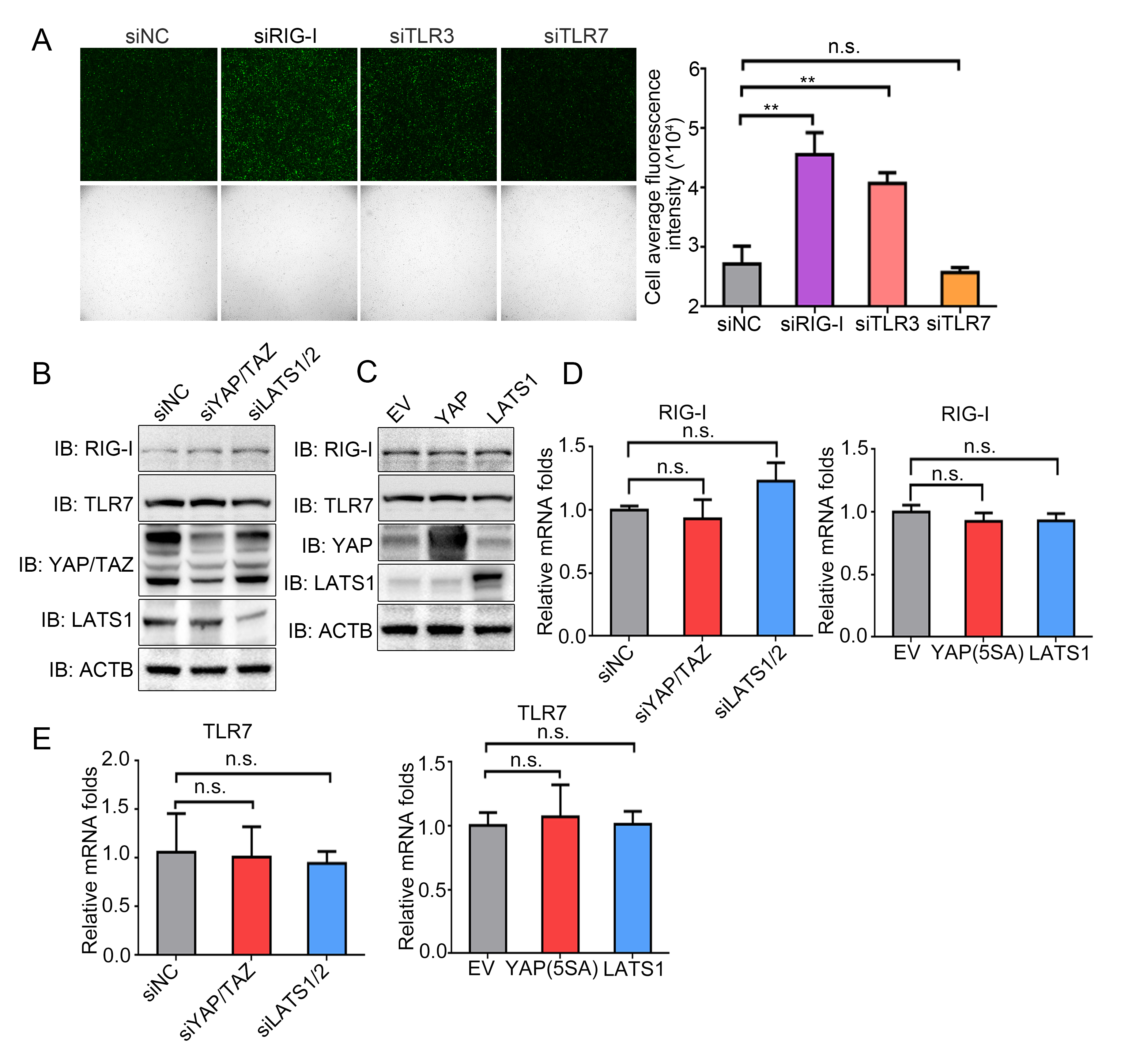

Supplement: S5 Fig — (A) A549 cells transfected with siNC, siRIG-I, siTLR3 and siTLR7 were infected with PR8-GFP. GFP fluorescence was detected (left). Cell average fluorescence intensity per cell was calculated (right). (B, C) The detection of indicated protein levels was conducted using immunoblotting in A549 cells with knockdown of YAP/TAZ and LATS1/2 (B), or overexpression of YAP(5SA) and LATS1 (C). (D, E) The mRNA expressions of RIG-I (D) and TLR7 (E) were detected upon YAP/TAZ and LATS1/2 knockdown, or YAP(5SA) and LATS1 overexpression. All of the experiments were repeated at least three times. Data in (A, D-E) are presented as means ± SD. *p < 0.05, **p < 0.01, ***p < 0.001. n.s., not significant (p > 0.05). (TIF) [file ppat.1010505.s005.tif]

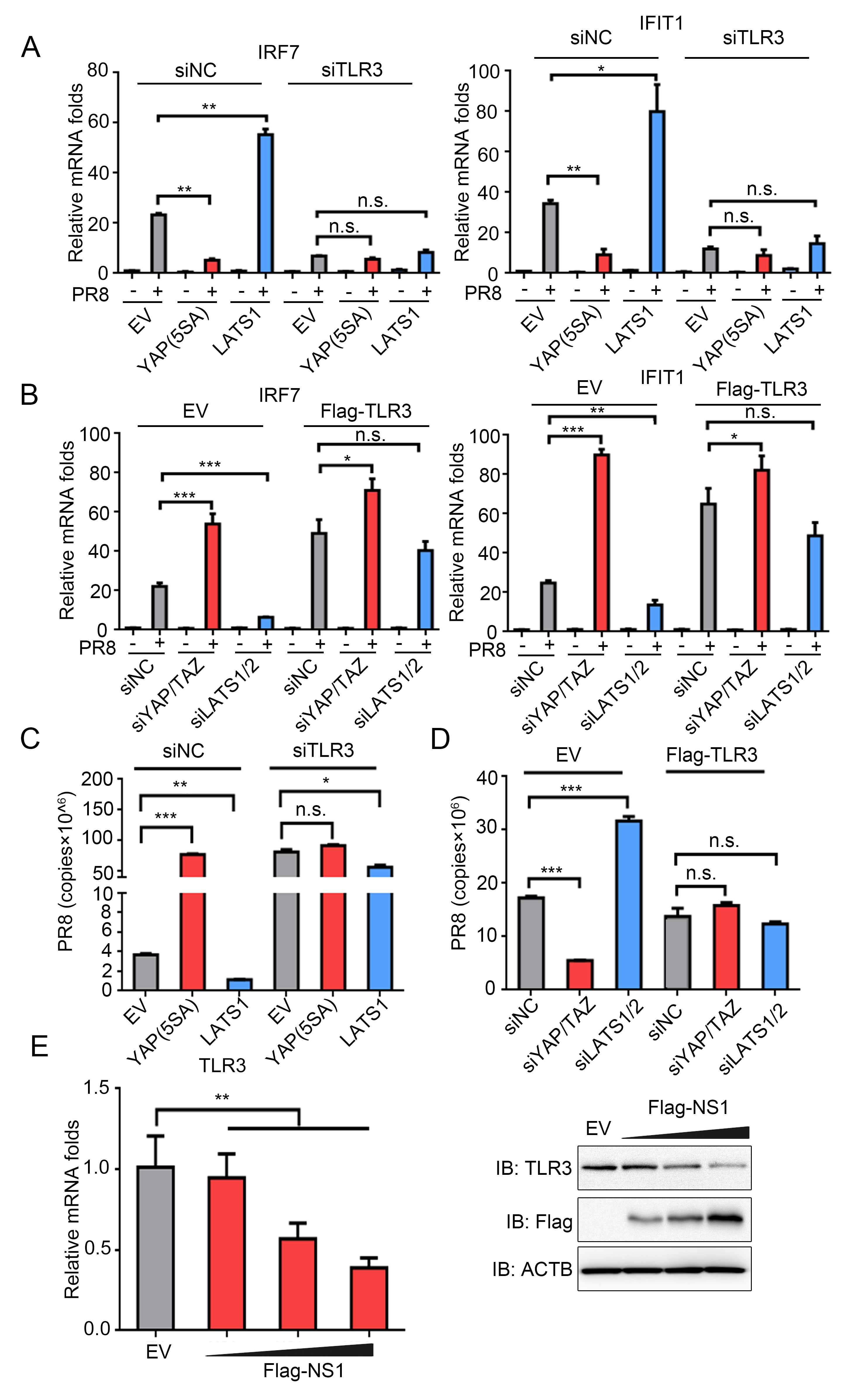

Supplement: S6 Fig — (A) EV-, YAP(5SA)-, and LATS1-overexpressing cells were transfected with siNC or siTLR3, and then infected with PR8 virus for 12 hours. IRF7 and IFIT1 expression levels were assessed by RT-qPCR. (B) siNC, siYAP/TAZ and siLATS1/2 were transfected into cells stably expressing EV or TLR3. PR8 was added to cells for 12 hours before IRF7 and IFIT1 expression levels were assessed by RT-qPCR. (C) EV-, YAP(5SA)-, and LATS1-overexpressing cells were transfected with siNC or siTLR3. Virus copies were measured. (D) Cells stably expressing TLR3 were transfected with NC scrambled siRNA and siRNAs targeting YAP/TAZ or LATS1/2. PR8 was added to cells for 12 hours before virus copies were measured. (E) Different amounts of Flag-NS1 were transfected into A549 cells. WCL was subjected to RT-qPCR (left) and immunoblotting (right) for measurement of TLR3 expression. All of the experiments were repeated at least three times. Data in (A-E) are presented as means ± SD. *p < 0.05, **p < 0.01, ***p < 0.001. n.s., not significant (p>0.05). (TIF) [file ppat.1010505.s006.tif]

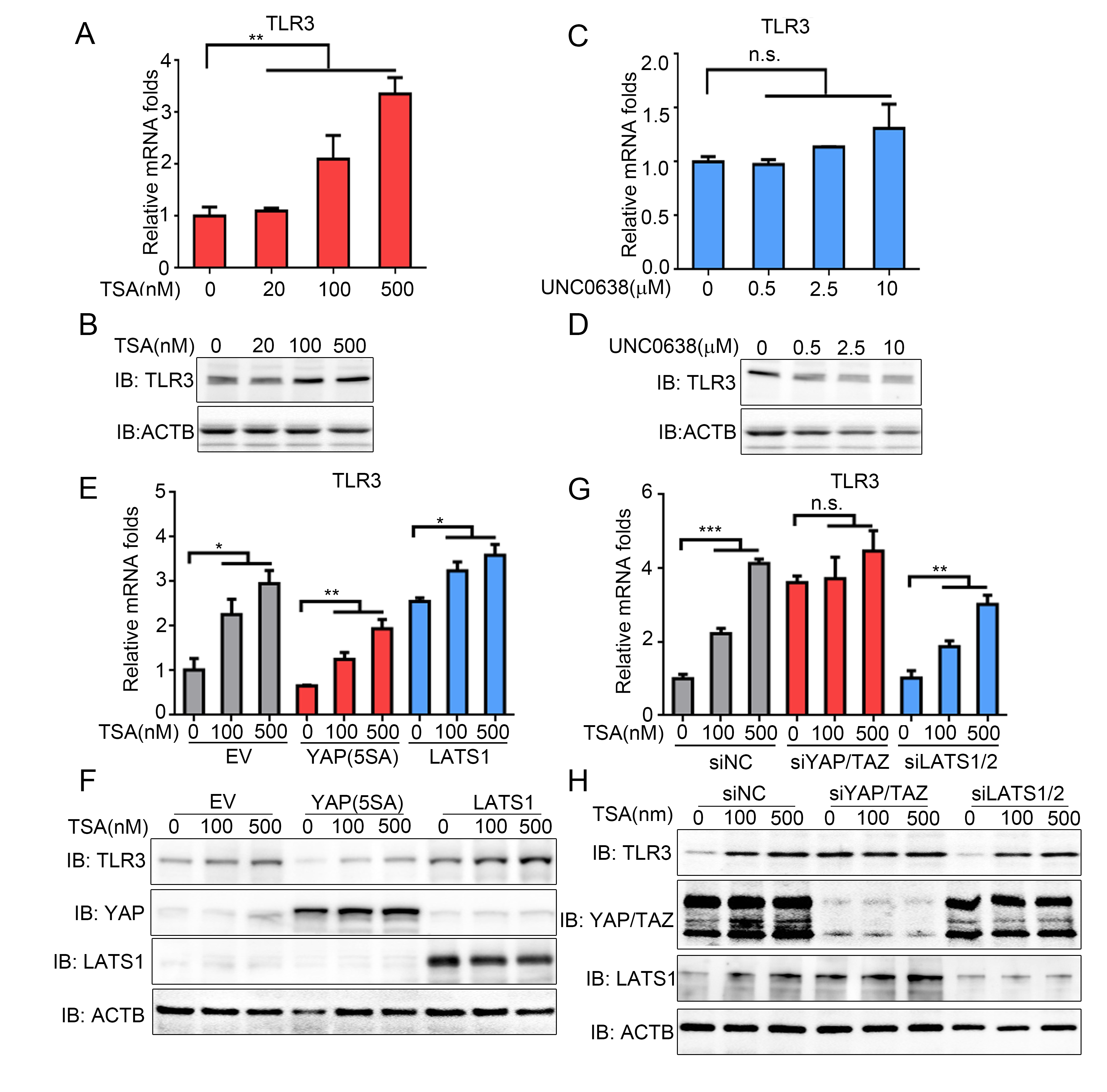

Supplement: S7 Fig — (A, B) Indicated concentrations of TSA were added to cells. Cells were then harvested and RT-qPCR for mRNA level (A) and immunoblotting for protein level (B) of TLR3 were conducted. (C, D) Cells were treated with UNC0638 before lysed for detection of mRNA level (C) and protein level (D) of TLR3. (E, F) A549 cells were transfected with YAP(5SA) and LATS1 (E) for 24 hours. Cells were then treated with TSA (100 and 500 nM) for another 6 hours before RT-qPCR analysis (E) and immunoblotting (F). (G, H) siNC, siYAP/TAZ and siLATS1/2 were transfected into A549 cells. TSA were treated before harvesting for RT-qPCR analysis (G) and immunoblotting (H). All of the experiments were repeated at least three times. Data in (A, C, E and G) are presented as means ± SD. *p < 0.05, **p < 0.01, ***p < 0.001. n.s., not significant (p>0.05). (TIF) [file ppat.1010505.s007.tif]
