# Supplementary material for: Influenza A virus NS1 protein hijacks YAP/TAZ to suppress TLR3-mediated innate immune response
Source: PLoS Pathog. 2022 May 3;18(5):e1010505. doi: 10.1371/journal.ppat.1010505 (PMC9122210; doi:10.1371/journal.ppat.1010505)
Supplement: S1 Table — (DOCX) [file ppat.1010505.s008.docx]

| **Oligos used in the study** |  |  |
| --- | --- | --- |
| **RNAi** | **Sequence** | **Species** |
| LATS1 | CCUCCAUACGAGUCAAUCAdTdT | Human |
| LATS2 | CAAGCAUCCUGAGCACGCAdTdT | Human |
| YAP | CCACCAAGCUAGAUAAAGAdTdT | Human |
| TAZ | CCGCAGGGCTCATGAGTATdTdT | Human |
| siTEADs | GAUCAACUUCAUCCACAAGCUdTdT | Human |
| siTLR3 | GGAAGAUAAUGAUAUUCCAGGdTdT | Human |
| siRIG-I | GUUGGAGGAGUAUAGAUUAdTdT | Human |
| siTLR7 | GACCUUGGAUCUAAGUAAAdTdT | Human |
| HDAC4 | CGACAGGCCUCGUGUAUGAdTdT | Human |
| HDAC6 | CUGCAAGGGAUGGAUCUGAdTdT | Human |
| HDAC7 | GGACAAGAGCAAGCGAAGUdTdT | Human |
| HDAC9 | GGAGAAGGGUACAAUAUAAdTdT | Human |
| HDAC11 | GGGCUACCAUCAUUGAUCUdTdT | Human |
|  |  |  |
| **Primers used for RT-qPCR** | **Sequence** | **Species** |
| CTGF forward primer | CTTGCGAAGCTGACCTGGAAGA | Human |
| CTGF reverse primer | CCGTCGGTACATACTCCACAGA | Human |
| CYR61 forward primer | GGAAAAGGCAGCTCACTGAAGC | Human |
| CYR61 reverse primer | GGAGATACCAGTTCCACAGGTC | Human |
| IFNB1 forward primer | CTTGGATTCCTACAAAGAAGCAGC | Human |
| IFNB1 reverse primer | TCCTCCTTCTGGAACTGCTGCA | Human |
| CXCL8 forward primer | GAGAGTGATTGAGAGTGGACCAC | Human |
| CXCL8 reverse primer | CACAACCCTCTGCACCCAGTTT | Human |
| IRF7 forward primer | CCACGCTATACCATCTACCTGG | Human |
| IRF7 reverse primer | GCTGCTATCCAGGGAAGACACA | Human |
| TLR3 forward primer | GCGCTAAAAAGTGAAGAACTGGAT | Human |
| TLR3 reverse primer | GCTGGACATTGTTCAGAAAGAGG | Human |
| TLR7 forward primer | CTTTGGACCTCAGCCACAACCA | Human |
| TLR7 reverse primer | CGCAACTGGAAGGCATCTTGTAG | Human |
| RIG-I forward primer | CACCTCAGTTGCTGATGAAGGC | Human |
| RIG-I reverse primer | GTCAGAAGGAAGCACTTGCTACC | Human |
| IFIT1 forward primer | GCCTTGCTGAAGTGTGGAGGAA | Human |
| IFIT1 reverse primer | ATCCAGGCGATAGGCAGAGATC | Human |
| YAP forward primer | TGTCCCAGATGAACGTCACAGC | Human |
| YAP reverse primer | TGGTGGCTGTTTCACTGGAGCA | Human |
| TAZ forward primer | GAGGACTTCCTCAGCAATGTGG | Human |
| TAZ reverse primer | CGTTTGTTCCTGGAAGACAGTCA | Human |
| LATS1 forward primer | CACTGGCTTCAGATGGACACAC | Human |
| LATS1 reverse primer | GGCTTCAGTCTGTCTCCACATC | Human |
| LATS2 forward primer | GTTCTTCATGGAGCAGCACGTG | Human |
| LATS2 reverse primer | CTGGTAGAGGATCTTCCGCATC | Human |
| TEAD1 forward primer | CCTGGCTATCTATCCACCATGTG | Human |
| TEAD1 reverse primer | TTCTGGTCCTCGTCTTGCCTGT | Human |
| GAPDH forward primer | GTCTCCTCTGACTTCAACAGCG | Human |
| GAPDH reverse primer | ACCACCCTGTTGCTGTAGCCAA | Human |
| HDAC4 forward primer | AGGTGAAGCAGGAGCCCATTGA | Human |
| HDAC4 reverse primer | GGTAGTTCCTCAGCTGGTGGAT | Human |
| HDAC6 forward primer | GCCTCAATCACTGAGACCATCC | Human |
| HDAC6 reverse primer | GGTGCCTTCTTGGTGACCAACT H | Human |
| HDAC7 forward primer | TCCTGGCACAGCGGATGTTTGT | Human |
| HDAC7 reverse primer | TGAAGGCGAGGTCAGTGACACT | Human |
| HDAC9 forward primer | TCTCGTCTCCAGGACTCACTCT | Human |
| HDAC9 reverse primer | GCACTGGTGTTTCAGCATCAAGG | Human |
| HDAC11 forward primer | CTTCTGTGCCTATGCGGACATC | Human |
| HDAC11 reverse primer | GAAGTCTCGCTCATGCCCATTG | Human |
| Ctgf forward primer | TGCGAAGCTGACCTGGAGGAAA | Mouse |
| Ctgf reverse primer | CCGCAGAACTTAGCCCTGTATG | Mouse |
| Cyr61 forward primer | GTGAAGTGCGTCCTTGTGGACA | Mouse |
| Cyr61 reverse primer | CTTGACACTGGAGCATCCTGCA | Mouse |
| Yap forward primer | CCAGACGACTTCCTCAACAGTG | Mouse |
| Yap reverse primer | GCATCTCCTTCCAGTGTGCCAA | Mouse |
| Ifnb1 forward primer | GCCTTTGCCATCCAAGAGATGC | Mouse |
| Ifnb1 reverse primer | ACACTGTCTGCTGGTGGAGTTC | Mouse |
| Irf3 forward primer | CGGAAAGAAGTGTTGCGGTTAGC | Mouse |
| Irf3 reverse primer | CAGGCTGCTTTTGCCATTGGTG | Mouse |
| Tlr3 forward primer | GTCTTCTGCACGAACCTGACAG | Mouse |
| Tlr3 reverse primer | TGGAGGTTCTCCAGTTGGACCC | Mouse |
| Il-6 forward primer | TACCACTTCACAAGTCGGAGGC | Mouse |
| Il-6 reverse primer | CTGCAAGTGCATCATCGTTGTTC | Mouse |
| Gapdh forward primer | CATCACTGCCACCCAGAAGACTG | Mouse |
| Gapdh reverse primer | ATGCCAGTGAGCTTCCCGTTCAG | Mouse |
| NP forward primer | AATAAGGCGAATCTGGCGCCAA | A/PR/8/34 |
| NP reverse primer | CATCCTGGGATCCATTCCGGT | A/PR/8/34 |
|  |  |  |
| **Primers used for ChIP-qPCR** | **Sequence** | **Species** |
| TLR3 P1 forward primer | CAACAATCATTTATTTGCTTGC | Human |
| TLR3 P1 reverse primer | TCCTCAGAATTCAAACCCTTG | Human |
| TLR3 P2 forward primer | TTAACTTGCAGAGGTCCAGACC | Human |
| TLR3 P2 reverse primer | CGTTTCAAACCGCCTCTG | Human |
| CYR61 forward primer | AGCAAACAGCTCACTGCCTT | Human |
| CYR61 reverse primer | ATGGTAGTTGGAGGGTCGTG | Human |
