# Supplementary material for: Influenza A virus NS1 protein hijacks YAP/TAZ to suppress TLR3-mediated innate immune response
Source: PLoS Pathog. 2022 May 3;18(5):e1010505. doi: 10.1371/journal.ppat.1010505 (PMC9122210; doi:10.1371/journal.ppat.1010505)
Supplement: S2 Table — (DOCX) [file ppat.1010505.s009.docx]

| **Antibodies used in the study** |  |  |  |  |  |  |  |
| --- | --- | --- | --- | --- | --- | --- | --- |
| **Antibody** | **Company** | **Catalog #** | **Species** | **Clone** | **Dilution** |  |  |
| **Primary Antibodies** |  |  |  |  | **WB** | **IF** | **ChIP** |
| anti-YAP/TAZ | CST | 8418 | Rabbit | Monoclonal | 1:1000 |  |  |
| anti-LATS1 | CST | 9153 | Rabbit | Monoclonal | 1:1000 |  |  |
| anti-GAPDH | HUABIO | M1211-1 | Rabbit | Polyclonal | 1:2000 |  |  |
| anti-Histone H3 | CST | 4499 | Rabbit | Monoclonal | 1:1000 |  |  |
| anti-ACTB | HUABIO | EM21002 | Mouse | Monoclonal | 1:10000 |  |  |
| anti-FLAG | HUABIO | 0912-1 | Rabbit | Polyclonal | 1:5000 |  |  |
| anti-p-YAP S127 | CST | 4911 | Rabbit | Polyclonal | 1:2000 |  |  |
| anti-p-LATS1 T1079 | CST | 8654 | Rabbit | Polyclonal | 1:2000 |  |  |
| anti-TLR3 | CST | 6961 | Rabbit | Monoclonal | 1:1000 |  |  |
| anti-TLR7 | CST | 5632 | Rabbit | Monoclonal | 1:1000 |  |  |
| anti-RIG-I | abcam | ab180675 | Rabbit | Monoclonal | 1:1000 |  |  |
| anti-IRF3 | CST | 4302 | Rabbit | Monoclonal | 1:1000 |  |  |
| anti-p-IRF3 S396 | CST | 4947 | Rabbit | Monoclonal | 1:1000 |  |  |
| anti-TEAD1 | CST | 12292 | Rabbit | Monoclonal | 1:1000 |  |  |
| anti-P38 | CST | 9212 | Rabbit | Monoclonal | 1:1000 |  |  |
| anti-p-P38 | CST | 9211 | Rabbit | Monoclonal | 1:1000 |  |  |
| anti-Influenza A virus NP | GeneTex | GTX125989 | Rabbit | Polyclonal | 1:5000 |  |  |
| anti-YAP1 | CST | 14074 | Rabbit | Monoclonal |  |  | 1:50 |
| anti-YAP1 | SIGMA | WH0010413M1 | Mouse | Monoclonal |  | 1:200 |  |
| anti-Histone H3 (acetyl K9 + K14 + K18 + K23 + K27) | abcam | ab47915 | Rabbit | Polyclonal | 1:5000 |  | 1:100 |
| DAPI | Invitrogen | D3571 |  |  |  |  |  |
|  |  |  |  |  |  |  |  |
| **Secondary Antibodies** |  |  |  |  |  |  |  |
| Goat anti-Rabbit IgG-HRP antibody | HUABIO | HA1001 |  |  | 1:10000 |  |  |
| Goat anti-Mouse IgG-HRP antibody | HUABIO | HA1006 |  |  | 1:10000 |  |  |
| anti-mouse IgG secondary Antibody, Alexa Fluor 594 | Thermo Fisher Scientific | R37115 |  |  |  | 1:2000 |  |
