# Supplementary material for: Influenza A virus NS1 protein hijacks YAP/TAZ to suppress TLR3-mediated innate immune response
Source: PLoS Pathog. 2022 May 3;18(5):e1010505. doi: 10.1371/journal.ppat.1010505 (PMC9122210; doi:10.1371/journal.ppat.1010505)
Supplement: Attachment — Submitted filename: rebuttal_letter.docx [file ppat.1010505.s010.docx]

**Response to Reviewer’s Comments**

**Influenza A virus NS1 protein hijacks YAP/TAZ to suppress TLR3-mediated innate immune response**

Qiong Zhang, Xujun Zhang, Xiaobo Lei, Hai Wang, Jingjing Jiang, Yucong Wang, Kefan Bi, Hongyan Diao

Submitted for review in PLOS Pathogens (Manuscript ID: PPATHOGENS-D-21-02312)

Overall, we thank the two reviewers for their service and constructive suggestions for this manuscript. We have now performed additional experiments and provided more detailed information to address all the concerns raised by the reviewers and revised our manuscript accordingly. We believe our revised manuscript could support our conclusion that NS1 protein of IAV suppresses TLR3 signaling through YAP/TAZ in a more comprehensive way. Our responses to the specific comments are listed below in blue font.

**Reviewer 1:**

**Major questions:**

1. The authors observed the IRF3 activation upon the activation of YAP (Fig 1F). This seems contradictory to the observation that deficiency of YAP resulted in elevated IRF3 activation (Fig 5F). If NS1 inhibited the expression of IRF3, why is IRF3 activated upon IAV infection?

We thank the reviewer for carefully reviewing the manuscript and pointing out this important issue. To clarify, upon IAV infection, the viral RNA that is present within infected cells is recognized as foreign by PRRs such as TLR3, TLR7 and RIG-I, which leads to the activation of the innate immune response, including the activation of IRF3 and further, the secretion of IFNs (Guillot et al., 2005; Lund et al., 2004; Pichlmair et al., 2006). Thus, it is conceivable that IRF3 is activated remarkably upon infection both in vitro and in vivo (Figs 1A and 1F). Of note, we focused on the anti-innate immunity role NS1 plays via YAP/TAZ during IAV infection in our present study. In support of our conclusion, we found the presence of NS1 activates YAP/TAZ, which in turn, blocks the TLR3 signaling. Thus, it is reasonable that YAP deficiency leads to enhanced IRF3 activation both in vitro and in vivo (Figs 5D and 5F). Together, we think it is not contradictory that IRF3 is activated upon IAV infection, while NS1 inhibits the activation of IRF3 via YAP/TAZ.

2. To avoid unnecessary confusion, the authors need to describe the protein constructs more accurately. For example, the residue numbers in YAP del C should be described (i.e., residues xx-xx). Other proteins should also be described the same way.

276-488

To make it clearer, we have added the description of TAD of YAP as “C-terminal transcriptional activation domain (TAD, residues 276-488) of YAP” on its first appearance in the revised manuscript (lines 192-193). In addition, the references from where the plasimids CTGF, GTIIC and CTGF-ΔTB luciferase reporter constructs derived were cited in Materials and Methods section (lines 561-565).

3. Ref 36 indicated that the C-terminal TAD of YAP is responsible for binding to HDAC7. Somehow, the authors did not connect the present results with the previous finding. For example, ref 36 should be mentioned in line 349, although they cited the paper later in the discussion section.

We thank the reviewer for pointing this out. The ref has been mentioned in proper position of our revised manuscript (lines 353-355).

4. Similarly, the authors did not cite ref 36 when describing their finding that NS1 binds to the C-terminal TAD of YAP (lines 184-192). This makes it difficult to understand the rationale of their test; i.e., why did the authors suddenly test the binding of NS1 and the C-terminal TAD? Citing the paper in the right place will help readers follow the authors’ rationale behind their experimental design.

We agree with the reviewer that test the binding of NS1 with the C-terminal TAD of YAP seemed abrupt without citation. We have added the reference in the corresponding sentence in our revised manuscript (lines 192-195).

5. Activation of YAP/TAZ can antagonize the function of TBK1 and IRF3. The authors also mentioned these previous findings. However, it is unclear whether the authors indicate that suppressing TLR3 transcription is the dominant mechanism by which NS1 interferes with immune responses. So, the authors need to clarify in the discussion section if they exclude other mechanisms.

We thank the reviewer for this important remark. To clarify, previous reports demonstrated YAP could prevent the activation of TBK1 and IRF3 by direct binding. However, we could not simply use knockdown or overexpression techniques to exclude these mechanisms by which YAP utilizes to block IAV-induced innate immune response, because these proteins are downstream TLR3, which are indispensable for TLR3 signaling activation. Instead, we provided evidences that either knockdown or exogenously expression of TLR3 could tremendously abrogate the regulation of type I IFN and inflammatory signalings by YAP/TAZ (new Figs 5G-5J, new S6A-S6D Figs), which indicated that TLR3 is crucial for YAP/TAZ-regulated innate immune response during IAV infection. We also have extended Discussion section regarding this (lines 440-445).

**Minor points:**

1. Lines 166 and 167; Figure 1E and Fig 1F should be swapped.

This mistake has been corrected.

2. Some references are not correctly formatted. For example, see ref 30.

The format of references has been checked and corrected.

**Reviewer 2:**

**General comments:**

For all of the experiments, the authors have used the mouse adapted flu strain PR8 to show the effect of NS1 on the effector proteins of the Hippo pathway. Although PR8 serves as an appropriate model (especially since the study uses mouse models) it would be useful to use other strains of flu to corroborate whether these findings are universal in flu infection. At least in the transfection experiments, NS1 from other flu strains should be included to check for binding to YAP/TAZ to confirm universal phenotypes.

We thank the reviewer for the helpful suggestion to improve the quality of our manuscript. Unfortunately, we were unable to obtain other strains of IAV. Therefore, we used NS1 proteins of A/California/07/2009 and A/Hong Kong/156/1997 to address whether their binding with YAP/TAZ are a universal phenotype by coimmunoprecipitation assay. The results have been shown in new Fig S2A. Indeed, NS1 proteins of these two strains are also capable of interaction with YAP/TAZ directly.

**Specific comments:**

In Fig 3, the authors indicate increased production of flu virus in cells constitutively expressing active YAP. This needs to be confirmed by measuring viral titres using plaque assays along with fluorescence measurements that the authors have performed. Increase in fluorescent punctae may not necessarily lead to production of infectious virions and it would be useful to determine whether this is the case or defective particles are produced. With the current set of experiments it is not clear which step in the viral lifecycle is facilitated by YAP/TAZ dependent immune regulation e.g. entry, replication, assembly or release.

As suggested by the reviewer, we performed plaque assays to confirm the proliferation of infectious virion is regulated by YAP/TAZ overexpression or knockdown. As shown in new Figure 3A and 3B, the viral titers were regulated by YAP/TAZ in the same way as fluorescent punctae presenting. We focused on the mechanisms underlying the suppression of antiviral innate immune response by YAP/TAZ in host cells in our current manuscript. Thus, as regards which step in the viral lifecycle is facilitated by YAP/TAZ, it is an interesting question to ask, though we think it is beyond the scope of this story.

Along the same lines, it is difficult to understand the magnitude of the effect in Fig 3B; there seems to be almost a complete block in virus production in the YAP-depleted cells based on the fluorescence data. From previous literature, the TLR3 signaling is thought to play a minor role in restricting infection compared to RIG-I. The authors should add some discussion on this point. It might be useful to have RIG-I ko/overexpressing cells as controls in this experiment. It would also be useful to check whether other pathways are affected, such as TLR7, to be able to quantify the magnitude of this phenotype.

We thank the reviewer for pointing this issue out. To clarify, previous reports suggested that YAP/TAZ inhibit the innate antiviral defense by preventing the K63-linked ubiquitination of TBK1 and disrupting its interaction with MAVS, STING and IRF3 (Zhang et al., 2017). YAP could also interact with IRF3, preventing its dimerization and nuclear translocation, which further reduces the production of IFN-β and ISGs in response to virus infection (Wang et al., 2017). Our current manuscript presented a new mechanism for YAP to antagonize the innate antiviral defense against IAV, by which YAP inhibits TLR3 expression. Thus, considering the crucial role YAP plays in regulating the innate immune response, we think it is not surprising that loss of YAP results in suppression of virus production predominantly.

As regards the role TLR3, RIG-I and TLR7 play in restricting IAV infection, we agree with the reviewer that RIG-I exerts profound effects on eliciting type I interferon signaling upon IAV infection. As recommended, RIG-I was also knocked down by siRNAs as a control in new Fig 3B. The results showed loss of RIG-I indeed increased IAV replication significantly. At the same time, we also knocked down TLR3 and TLR7 and found knockdown of TLR3 could significantly enhance the replication of virion. However, TLR7 knockdown seemed barely having any effect on the replication of virion (new S5A Fig). Thus, we reasoned RIG-I and TLR3, but not TLR7 play important roles in restricting virus replication upon IAV infection in A549 cells, which was also supported by previous report that RIG-I and TLR3, but not TLR7 are responsible for IFN induction by influenza virus in human lung alveolar epithelial cells (Wu et al., 2015). More work is needed in the future to address whether TLR7 is needed for triggering IFN signaling in other cell types in the lung during IAV infection. In short, the roles of different PRRs play during IAV infection are still controversial and need further investigation. We also have extended our discussion on this issue (lines 464-473).

In Fig 4, dsRNA or Poly I:C controls should be included in the siRNA knockdown experiments to measure IFN and other cytokine responses to delineate effects of other viral components that might perturb this pathway. Transcriptional expression/repression of target genes should be recapitulated in these controls.

As suggested by the reviewer, we have reconducted the siRNA knockdown experiments and the treatment of poly I:C was used as a control. As shown in new Figure 4A, the induction of antiviral and proinflammatory factors indicates poly I:C exerts a similar magnitude of effect with PR8 virus upon siRNAs knockdown.

In Fig 5, the increase in immune signaling proteins is clear from the YAP dose-dependent experiment. It is however not clear that this arises from TLR3 signaling. Expression of other signaling pathways e.g. TLR7 should also be measured. Does KD/overexpression of TLR3 correlate negatively with YAP phenotype?

In order to confirm our conclusion that TLR3 mediates YAP/TAZ-regulated innate immune signaling, we knocked down TLR3 in EV-, YAP(5SA)- and LATS1-overexpresing cells, the results showed TLR3 knockdown significantly abrogated the stimulation of IRF3 and p38 signalings and promoted virus replication in all these cells to similar levels upon IAV infection. (new Figs 5G and 5H, new S6A and S6C Figs). Consistently, TLR3 overexpression resulted in strengthening IRF3 and p38 signalings and reducing virus copies in cells with knockdown of YAP/TAZ, LATS1/2 or NC to a similar level (new Fig 5I-5J, new S6B and S6D Figs). We believe all these experiments could further support the conclusion we made.

As regards the role of TLR7 plays, firstly, as mentioned above, we found TLR7 does not participate in restraining virus replication in A549 cells. Secondly, we measured the expression of TLR7 and RIG-I in both YAP/TAZ, LATS1/2 knockdown cells and YAP, LATS1 overexpression cells. The results showed neither TLR7 nor RIG-I was regulated by YAP/TAZ (new S5B-S5E Figs). Thus, we concluded that TLR3 is responsible for YAP/TAZ-regulated innate immune signaling during IAV infection.

**Minor points:**

There are also several grammatical and typographical errors in the current version of the manuscript, which needs to be corrected.

We have gone through the whole manuscript and corrected these errors.

**References**

Guillot, L., Le Goffic, R., Bloch, S., Escriou, N., Akira, S., Chignard, M., and Si-Tahar, M. (2005). Involvement of toll-like receptor 3 in the immune response of lung epithelial cells to double-stranded RNA and influenza A virus. The Journal of biological chemistry *280*, 5571-5580.

Lund, J.M., Alexopoulou, L., Sato, A., Karow, M., Adams, N.C., Gale, N.W., Iwasaki, A., and Flavell, R.A. (2004). Recognition of single-stranded RNA viruses by Toll-like receptor 7. Proceedings of the National Academy of Sciences of the United States of America *101*, 5598-5603.

Pichlmair, A., Schulz, O., Tan, C.P., Näslund, T.I., Liljeström, P., Weber, F., and Reis e Sousa, C. (2006). RIG-I-mediated antiviral responses to single-stranded RNA bearing 5'-phosphates. Science *314*, 997-1001.

Wang, S., Xie, F., Chu, F., Zhang, Z., Yang, B., Dai, T., Gao, L., Wang, L., Ling, L., Jia, J.*, et al.* (2017). YAP antagonizes innate antiviral immunity and is targeted for lysosomal degradation through IKKvarepsilon-mediated phosphorylation. Nature immunology *18*, 733–743.

Wu, W., Zhang, W., Duggan, E.S., Booth, J.L., Zou, M.H., and Metcalf, J.P. (2015). RIG-I and TLR3 are both required for maximum interferon induction by influenza virus in human lung alveolar epithelial cells. Virology *482*, 181-188.

Zhang, Q., Meng, F., Chen, S., Plouffe, S.W., Wu, S., Liu, S., Li, X., Zhou, R., Wang, J., Zhao, B.*, et al.* (2017). Hippo signalling governs cytosolic nucleic acid sensing through YAP/TAZ-mediated TBK1 blockade. Nature cell biology *19*, 362-374.
